# User embracement in the Family Health Strategy in a city in the north of Minas Gerais, Brazil: a descriptive study, 2019-2020

**DOI:** 10.1590/S2237-96222022000200019

**Published:** 2022-10-03

**Authors:** Samara Frantheisca Almeida Barbosa, Paula Rayane Calixto, Renatha Priscilla Ferreira da Silva, Edmar Rocha Almeida

**Affiliations:** 1 Universidade Estadual de Montes Claros, Programa de Pós-Graduação em Cuidado Primário em Saúde, Montes Claros, MG, Brazil; 2 Secretaria Municipal de Saúde de Fruta de Leite, Fruta de Leite, MG, Brazil; 3 Associação Paulista para o Desenvolvimento da Medicina, Saúde Indígena, Canarana, MT, Brazil; 4 Secretaria Municipal de Saúde de Taiobeiras, Programa de Residência Multiprofissional em Saúde da Família e Comunidade, Taiobeiras, MG, Brazil

**Keywords:** User Embracement, International Classification of Primary Care, Family Health Strategies, Health Profile, Health Information Systems

## Abstract

**Objective:**

To analyze the user embracement records of the Family Health Strategy (FHS) teams in a city in the North of Minas Gerais, Brazil.

**Methods:**

This was a cross-sectional and descriptive study based on secondary data. We analyzed FHS nurse and pharmacist service user embracement records for the period from March 2019 to February 2020. The total number of user embracement records and total number of records per service user were calculated according to the codes used by the International Classification of Primary Care (ICPC-2).

**Results:**

A total of 20,513 user embracement records were identified, the majority for female health service users (63.5%), those aged between 20 and 59 years (52.5%) and related to the ICPC-2 Procedure chapter (18.5%). User embracement activities were carried out for only 31.1% of the population resident in the FHS territories.

**Conclusion:**

Having knowledge about user embracement in the FHS and the main reasons for it highlights the importance of interventions targeting prevalent groups, in addition to contributing to the organization of health care.

**Study contributions**

**Main results**

The user embracement activities provided in the FHS services in Taiobeiras, MG, between March 2019 and February 2020, mostly to female and adults - 31.1% of residents in the FHS territories -, had between one and five reasons per record, in particular diseases found in the ICPC-2 musculoskeletal and respiratory chapters.

**Implications for services**

This study can improve public policies for the groups that most use the FHS, as well as demonstrating the resolutive capacity of the service, in addition to enhancing use of the ICPC-2 to provide better characterization of demand for services.

**Perspectives**

The study points to the need for further research on the subject, aimed at filling gaps that still exist using more robust analysis.

## Introduction

In Brazil, the National Health System (*Sistema Único de Saúde* - SUS) is based on primary health care, this being the level of care responsible for organizing care directed at the health needs of the population.^1^ The implementation and expansion of the Family Health Strategy (FHS) has structured primary health care and improved people's access to health services.^2^ ^,^ ^3^ Primary health care is characterized by four essential attributes: first contact access, longitudinality, comprehensiveness and care coordination.^4^ To operationalize first contact access in the FHS, the Ministry of Health recommends user embracement of spontaneous demand in primary care. Provision of this service implies the setting up of mechanisms to listen to the population, avoiding care being restricted to certain groups.^5^ ^,^ ^6^

The nature of demand for services in primary health care is complex and varied, involving bureaucratic issues, fears and psychological discomfort, in addition to the signs and symptoms of diseases that lead people to seek care in health services.^1^ Therefore, it is appropriate to use the International Classification of Primary Care, currently in its second version (ICPC-2), designed to classify problems related to people rather than diseases.^7^ The official FHS information system, in force since 2013, known as “e-SUS Atenção Primária” (e-SUS/APS), is structured according to the coding of the three ICPC-2 care record components: the reason for the consultation, diagnosis and process of care (intervention).^8^ Proper registration and coding of the reason for consultation are essential, as they allow health teams to identify service users” main demands and plan people's access to primary health care.^7^ ^,^ ^9^

The demand for health services can be influenced by individual, health-related, and sociodemographic characteristics, the epidemiological profile of the population, and the organization of health care availability.^10^ As such, first contact access is considered an indispensable attribute of primary health care, since, through access to services, it is possible to effect the comprehensiveness, longitudinality and the coordination of care offered by the SUS.^2^ However, part of the studies on spontaneous demand in Brazil evaluate access to health care from the perspective of medical care of spontaneous demand and not from the process of user embracement.^9^ ^,^ ^11^ Furthermore, the literature highlights the importance of understanding the ICPC-2 for the work of health teams in caring for service users and ensuring their embracement at their first contact.^9^ ^,^ ^11^ There is therefore an emerging need to know and analyze the profile of service users and their health problems that make up spontaneous demand for primary health care, from the perspective of their embracement through qualified listening, even if in specific locations. This information can help SUS professionals and managers to define strategies for organizing and setting the size of FHS teams, with the aim of reducing inequities in the population's access to health care.

The objective of this study was to analyze Family Health Strategy team user embracement records in a city in the north of the state of Minas Gerais, Brazil.

## Methods

This was a cross-sectional study conducted in the municipality of Taiobeiras, Minas Gerais, using data extracted from FHS user embracement records for the period from March 2019 to February 2020.

Taiobeiras is the headquarters of a health region located in the north of Minas Gerais, in the Alto Rio Pardo region. The Brazilian Institute of Geography and Statistics (IBGE) estimated the municipality's population to be 34,653 in 2021, 18.94% of whom lived in rural areas.^12^ In 2019, the local *per capita* gross domestic product (GDP) was R$ 13,843.51; and the human development index was 0.670. At the time of this study, the municipality's health network had 15 FHS centers, three of which are reference services for the rural population.^11^ In order to preserve the confidentiality of the information, the original FHS center names were given the names of colors: Blue, Red, Yellow, Pink, Black, White, Gray, Green, Purple, Brown, Orange, Beige, Gold, Violet and Bordeaux.

In the municipality, user embracement is performed essentially by nurses, but also by pharmacists in health centers where there is Multiprofessional Residency in Family and Community Health (five FHS centers during the data collection period). Occasionally, nursing assistants/technicians take on this role. All the health professionals mentioned here are trained to comply with the user embracement protocol which includes risk classification and vulnerability in the municipality.

During user embracement, appointments are scheduled and internal referrals are made on the day (between FHS professionals), either to specialized services or to urgency and emergency services, in which guidance is given and qualified listening is offered, with risk and vulnerability classification. This study included data on “initial listening/guidance”, corresponding to the care provided to people with complaints or signs/symptoms that result in internal referral on the day.

The data that served as the basis for this analysis were extracted from the municipality's FHS information system on July 8, 2020; these are the records of spontaneous demand, made by nurses and pharmacists of the FHS centers in Taiobeiras. The information is input to the system by the professional responsible for providing care, using the “individual care form” that meets the e-SUS/APS standard and is used exclusively by senior FHS professionals.^9^ The number of registered service users per FHS was also extracted from the system in order to identify the population present/resident in the territory of a given FHS.^3^

The study variables were: FHS center; number of consultations per FHS center; resident population per FHS; age of the service user; academic qualification of the professional providing care (nurse; pharmacist); service user sex (male; female); health problem/condition assessed (ICPC-2, primary and secondary); month in which user embracement was provided; and day of the week on which user embracement was provided. Some data were reorganized in two new categories: age group (in years: 0-9; 10-19; 20-59; 60 or more); and reasons for consultations, according to the ICPC-2 component/chapter to which they corresponded. 

Duplicate records or typing errors, such as services not performed by the FHS nurse or pharmacist, or not classified as user embracement, such as continuing care consultations (childcare or prenatal), were identified and manually corrected by the researchers using Microsoft Excel® (2010) spreadsheets. 

The total number of user embracement provided and the number of user embracement according to variable categories used in the study were calculated based on individual user data, person attended to, number of service users who benefited from user embracement at least once, and the number of records per service user. The proportion of primary health care user embracement, per FHS center, by months of the year and days of the week, was also calculated. We described the absolute number and percentage of complaints/signs and symptoms that led to the user embracement consultation.

The data were organized on Excel spreadsheets, as mentioned above, and were analyzed using the Statistical Package for Social Science® (SPSS) for Windows, version 18.0. 

The research project was approved by the Universidade Estadual de Montes Claros Research Ethics Committee on June 21, 2020, as per Opinion No. 4.101.307/2020.

## Results

A total of 31,610 user embracement records were extracted from the system and, following manual checking, a total of 20,513 records in Taiobeiras FHS centers remained. This is the total of records, regardless of the number of times a service user received care in the period analyzed, i.e. from March 2019 to February 2020.

The FHS centers with the highest number of user embracement records were Bordeaux (10.1%), followed by Violet (9.6%) and Gold (9.2%), while the center with the fewest records was Blue (2.9%). Of the total number of user embracement, 13,034 (63.5%) related to female service users and 7,479 (36.5%) were male; this higher proportion of female service users was found in each of the FHS centers, with this percentage varying between 59.6% (FHS Blue) and 68.8% (FHS Red) (Table 1).

**Table 1:** Distribution of the Primary Care user embracement records (n = 20,513), according to Family Health Strategy centers, Taiobeiras, Minas Gerais, Brazil, March 2019 to February 2020

Family Health Strategy Center	User embracement records			Users	
	Total	Sex		Resident population (% of the total population)	Population using the service (% of the resident population)
		Male n (%)	Female n (%)		
Blue^a^	592 (2.9)	239 (40.4)	353 (59.6)	1,385 (4.3)	399 (28.8)
Red	815 (4.0)	254 (31.2)	561 (68.8)	2,260 (7.0)	583 (25.8)
Yellow	882 (4.3)	320 (36.3)	562 (63.7)	2,056 (6.4)	571 (27.8)
Pink	925 (4.5)	338 (36.5)	587 (63.5)	1,928 (6.0)	590 (30.6)
Black	926 (4.5)	369 (39.9)	557 (60.1)	2,214 (6.9)	621 (28.0)
White^a^	1,151 (5.6)	450 (39.1)	701 (60.9)	2,248 (7.0)	701 (31.2)
Gray	1,287 (6.3)	504 (39.2)	783 (60.8)	2,201 (6.9)	736 (33.4)
Green	1,288 (6.3)	487 (37.8)	801 (62.2)	2,322 (7.2)	824 (35.5)
Purple^a^	1,468 (7.1)	526 (35.8)	942 (64.2)	2,106 (6.6)	820 (38.9)
Brown	1,718 (8.4)	637 (37.1)	1,081 (62.9)	2,171 (6.8)	907 (41.8)
Orange	1,736 (8.5)	588 (33.9)	1,148 (66.1)	2,110 (6.6)	886 (42.0)
Beige	1,788 (8.7)	617 (34.5)	1,171 (65.5)	2,218 (6.9)	919 (41.4)
Gold	1,887(9.2)	715 (37.9)	1,172 (62.1)	1,869 (5.8)	990 (53.0)
Violet	1,980 (9.6)	670 (33.8)	1,310 (66.2)	1,926 (6.0)	1,006 (52.2)
Bordeaux	2,070 (10.1)	765 (37.0)	1,305 (63.0)	3,068 (9.6)	1,039 (33.9)
Total	20,513 (100.0)	7,479 (36.5)	13,034 (63.5)	32,082 (100.0)	11,592 (36.1)

a) Teams caring for rural populations.

In the selected period, 11,592 users were provided with user embracement at their FHS center at least once, corresponding to 36.1% of the total resident population. The FHS center with the highest proportion of resident people in their territories - and the highest proportional number of people provided with user embracement by them - were the Gold (53.0%) and the Violet (52.2%); and the Red ESF (25.8%) was the one with the lowest proportion (Table 1).

The majority of the service users were between 20 and 59 years old (52.5%), followed by those aged 60 or older (24.7%), 0-9 years old (13.6%) and 10-19 years old (9.2%). A total of 17,120 (83.5%) user embracement were provided by nurses, while 3,393 (16.5%) were provided by pharmacists (Table 2). The proportion of female service users was higher in all age groups, except those under 9 years old, among whom males predominated (52.8%) (Table 2).

**Table 2:** Distribution of Family Health Strategy user embracement records (n = 20,513), according to characteristics of the service, service users and episode, Taiobeiras, Minas Gerais, Brazil, March 2019 to February 2020

Age group (in years)	User embracement records				Users			
	Health professional providing user embracement		Sex		Number of reasons per record (n = 20,412)		Number of records per service user in the period studied (n = 11,592)	
	Nurse (%)	Pharmacist (%)	Male (%)	Female (%)	1 (%)	2 or more (%)	1-3 (%)	4 or more (%)
≤ 9	2,261 (81.2)	525 (18.8)	1,470 (52.8)	1,316 (47.2)	2,055 (73.8)	731 (26.2)	1,542 (93.3)	111 (6.7)
10-19	1,529 (80.8)	363 (19.2)	791 (41.8)	1,101 (58.2)	1,465 (77.6)	422 (22.4)	1,234 (96.1)	50 (3.9)
20-59	8,994 (83.4)	1,784 (16.6)	3,246 (30.1)	7,532 (69.9)	8,414 (78.7)	2,277 (21.3)	5,695 (92.4)	470 (7.6)
≥ 60	4,336 (85.7)	721 (14.3)	1,972 (31.0)	3,085 (61.0)	3,664 (72.6)	1,384 (27.4)	2,155 (86.5)	335 (13.5)
Total	17,120 (83.5)	3,393 (16.5)	7,479 (36.5)	13,034 (63.5)	15,598 (76.4)	4,814 (23.6)	10,626 (91.7)	966 (8.3)

The number of reasons for encounter, as per ICPC-2, ranged from one to five per record/user embracement, and most occurred for only one reason (76.4%) (Table 2). The categorization of the reasons for encounter according to ICPC-2 codes resulted in the loss of 101 records, because they were registered as per ICD-10. The annual number of user embracement per user ranged from one to 14, with a higher frequency of one to three embracement per year (91.7%), with the majority (63.3%) requiring only one embracement per year.

**Figure 1:**
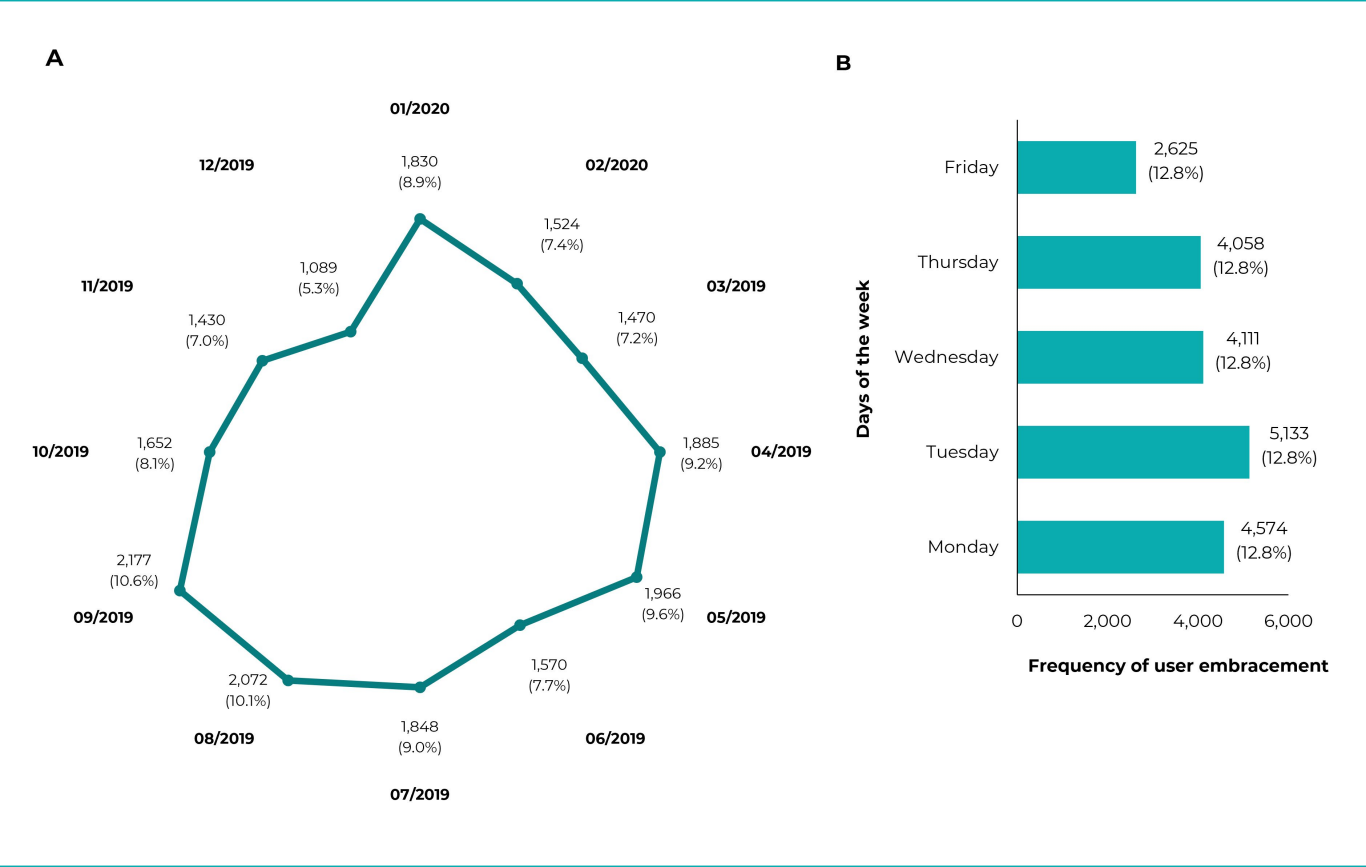
Distribution of the Family Health Strategy Primary Care user embracement records (n = 20,513), by months of the year (A) and days of the week (B) analyzed, Taiobeiras, Minas Gerais, Brazil, March 2019 to February 2020

Figure 1 shows the distribution of the frequency of the user embracement records according to month of the year and day of the week. The months with the highest number of user embracement records were September (10.6%) and August (10.1%) (Figure 1A). The highest frequencies of user embracement occurred on Tuesdays (25.0%), followed by Mondays (22.3%), Wednesdays (20.0%), Thursdays (19.8%) and Fridays (12.8%) (Figure 1B). 

**Table 3:** Distribution of the Family Health Strategy user embracement records (n = 25,767), according to International Classification of Primary Care component/chapter, Taiobeiras, Minas Gerais, Brazil, March 2019 to February 2020

ICPC-2 chapter^a^	Number of reasons (%)
Procedures (-)	4,761 (18.5)
Musculoskeletal (L)	3,168 (12.3)
Respiratory (R)	3,127 (12.1)
Digestive (D)	2,556 (9.9)
General and unspecified (A)	2,516 (9.8)
Circulatory (K)	2,094 (8.1)
Neurological (N)	1,669 (6.5)
Skin (S)	1,093 (4.3)
Psychological (P)	873 (3.4)
Endocrine/metabolic and nutritional (T)	796 (3.1)
Female genital (X)	682 (2.6)
Hearing (H)	659 (2.6)
Pregnancy, child-bearing, family planning (W)	604 (2.3)
Urological (U)	528 (2.0)
Eye (F)	374 (1.5)
Male genital (Y)	222 (0.9)
Blood, blood-forming organs and immune mechanism (spleen, bone marrow (B)	39 (0.1)
Social problems (Z)	6 (-)
Total	25,767 (100.0)

a) ICPC-2: International Classification of Primary Care.

The health conditions (n = 25,767) that gave rise to the user embracement were classified according to the ICPC-2 chapters (Table 3). The highest frequency of user embracement corresponded to the chapter on procedures (18.5%), followed by the chapters on musculoskeletal disorders (12.3%), respiratory disorders (12.1%) and digestive disorders (9.9%). Table 4 shows the ten most frequent ICPC-2 titles, which together accounted for 40.2% of the reasons for user embracement provided by the Taiobeiras FHS centers.

**Table 4:** Distribution of the main Family Health Strategy user embracement records (n = 10,357) according to International Classification of Primary Care title, Taiobeiras, Minas Gerais, Brazil, March 2019 to February 2020

ICPC-2^a^ title (code)	Number of reasons (%)
Consultation with primary care provider (-46)	2,038 (7.9)
Cough (R05)	1,488 (5.8)
Results examination/test/record/letter from other provider (-61)	1,219 (4.7)
Hypertension, complicated (K87)	1,202 (4.6)
Headache (N01)	953 (3.7)
No disease (A97)	951 (3.7)
Throat symptoms/complaint (R21)	671 (2.6)
Low back symptoms/complaint (L03)	660 (2.6)
Abdominal pain/cramps, general (D01)	616 (2.4)
Diabetes, non-insulin dependent (T90)	559 (2.2)
Genital feminino (X)	682 (2.6)
Total	10,357 (40.2)

a) ICPC-2: International Classification of Primary Care.

## Discussion

The study showed that during the period analyzed Bordeaux FHS center had the highest proportion of user embracement records in Taiobeiras, and the highest number of embracement among female users out of all the FHS centers. Less than half of the total population resident in FHS territories underwent at least one embracement at their FHS centers, and the number of times the same user required provision of embracement ranged from one to 14. As for age group, there was a higher frequency of care at the local FHS centers for individuals aged 20 to 59 years, mostly provided by nurses. The day of the week with the highest demand for care was Tuesday, while September was the month with the highest proportion of user embracement. Regarding the frequency of the reasons for user embracement provision per record, they varied from one to five types, among the reasons listed in the ICPC-2. The highest frequency was found for procedures, followed by musculoskeletal disorders, as per ICPC-2.

The variations in the profile of health service utilization, according to the FHS center, reflect the different demographic and socioeconomic characteristics among the coverage areas. The center with the highest absolute number of user embracement records (Bordeaux) serves the largest territorial population in the municipality, while the next highest centers (Violet and Gold) have populations of high social vulnerability. 

We found that approximately one third of the municipality's population resident in FHS territories was provided with user embracement by FHS centers, and most of them (63.3%) required a single consultation. However, 8.3% of the population that sought primary health care generated 4,632 (20.5%) user embracement records. This phenomenon, called “overutilization”, can be attributed to service users who need care or who overuse primary health care services. Furthermore, it demonstrates the need to discuss the resolutive capacity of the service through the incorporation of more effective practices, with more demand for care, such as the person-centered clinical method.^1^

The higher proportion of spontaneous demand among female service users corroborates the results of previous studies conducted in three FHS centers in Betim, Minas Gerais, primary care centers in Florianópolis, Santa Catarina, a primary care center in São Carlos, São Paulo, and another primary care center in Fortaleza, Ceará.^9^ ^,^ ^11^ ^,^ ^13^ ^,^ ^14^ Theoretically, women being less included in formal employment, their greater perception of diseases and symptoms and their better adherence to preventive measures are factors that increase the demand for health services among women.^14^ In contrast, male demand for outpatient care is mainly related to work or social security. There is evidence that males avoid health care spaces, are averse to prevention and self-care, and commonly delay seeking care.^15^

There was a higher proportion of adult users aged 20 to 59 years among those provided with user embracement in primary health care during the study period. There is no consensus in the literature about the frequency of demand for primary health care services among different age groups, although the studies we consulted point to a predominance of services provided to the adult population. The survey on demand for care carried out in the Fortaleza FHS center, mentioned in the previous paragraph, found prevalence of users between 41 and 60 years old.^14^ The study conducted in 2021 in São Carlos, in the state of São Paulo, also cited above, showed a predominance of adults in the 20-50 age range.^13^ Analysis of medical consultation records in a Fortaleza FHS center in 2015 showed that most users were 20 to 39 years old,^16^ while in primary care centers in Florianópolis, the study conducted in 2009 and also mentioned above found that service users were predominantly aged between 25 and 44 years old.^11^

In our study, we found a lower proportion of user embracement provided by pharmacists, since in the period investigated, only five of the 15 FHS centers had pharmacists in their teams. It is noteworthy that the inclusion of pharmacists in providing primary health care user embracement is in accordance with the guidelines for health service residency, namely: the development of new practices in health services and the improvement of health worker skills.^17^ A study carried out at a multiprofessional residency health center in Itajaí, Santa Catarina, found that service users provided with embracement by pharmacists assessed their care as being humanized, with their health needs being met, as well as exchange of experiences, which enabled linkage between these service users and the health professionals who assisted them.^18^

There was a higher proportion of user embracement on Tuesdays, followed by Mondays, and less demand on the other days of the week. The study carried out in Betim showed that demand for consultations is higher there on Mondays and Fridays.^9^ As for the months of the year, there was greater demand between August and September (winter). In Florianópolis primary care centers, it was found that the main demands are constant throughout the year, although there is an increase in the frequency of care in winter, due to coughs.^11^

With regard to the reasons for user embracement provision, we found a greater number of the ICPC-2 components related to diagnostic and preventive procedures, predominant among which were “Consultation with a primary care provider (-46)” and “Results examination/test/record/letter from another provider (-61)”. The procedure component relates to service user requirements for treatment, care instructions in the form of guidance given by health professionals, procedures and medication. However, this finding may reflect health professional difficulties in identifying a more adequate code to characterize the demand.^7^ A nationwide study on the reasons for medical care in primary health care identified that procedures such as prescription renewal and examination review are quite frequent.^19^

The musculoskeletal, respiratory and digestive problems that ranked second, third and fourth on the list of reasons for user embracement provision, respectively, were partially in agreement with data from other surveys. The study on Florianópolis primary care centers placed the circulatory chapter in third place on this list.^11^ In the FHS studies in Betim and Fortaleza, mentioned above, there were also differences in the order of respiratory, digestive and musculoskeletal conditions.^9^ ^,^ ^16^

The reasons for user embracement provision classified as falling under the General chapter (unspecified signs and symptoms, such as fever, generalized pain and fatigue) came in fifth place in our study, and it should be mentioned that it has high prevalence in most of the studies we consulted.^9^ ^,^ ^16^ There are health issues in primary care that are not subject to specific diagnosis, which gives a unique characteristic to primary care practice, namely active observation or acceptable delay.^1^ ^,^ ^9^ ^)^

The three most frequent reasons for provision of user embracement of a clinical nature were cough, followed by hypertension with complications and headache. There is no consensus in the literature as to the reasons for encounter coded according to ICPC-2. A national study identified preventive medicine/health maintenance, pregnancy and uncomplicated hypertension as the main reasons for medical consultations in primary health care.^19^ The study conducted in three FHS centers in Betim found headache, fever and cough to be predominant complaints during provision of user embracement.^9^ The study involving the FHS center in Fortaleza based on medical consultation records, found pregnancy, headache and preventive medicine/health maintenance to be most predominant,^16^ while in Florianópolis primary care centers the most frequent reasons for consultations were preventive medicine/health maintenance, cough and procedures (medication/prescription/renewal/injection).^11^

An important finding of this research was the fact that ten ICPC-2 titles corresponded to about 40% of the reasons for user embracement. In the aforementioned national study,^19^ seven reasons accounted for 50% of primary health care medical consultations, when administrative demands were included.

Although ICPC-2 is a broad classification of the main reasons for encounter in primary health care, it does have limitations. Some consultation data, such as classification of therapies, medications, physical examination results or complementary exams, are not codifiable. Thus, coding of the reason for encounter depends directly on health professionals having been trained to do it.^16^

A second limitation of the study was the fact that it excluded user embracement provided by nursing assistants/technicians, these being health professionals who did not use the “individual care form” on the information system. In addition, given the technical limitations in extracting data from the system, it was not possible to include “consultations on the day” in the analysis, because the volume of information was high and was limited to extraction of data on “initial listening/guidance”. Thus, the exclusion of these appointments made it impossible to conclude whether all primary health care services were represented, since the study analyzed only spontaneous demand. However, the volume of data was large, thus allowing inferences to be made.

The ICPC-2 is known to be considered the most appropriate tool for classifying the reasons for consultation in primary health care and thus assessing demand for consultation in accordance with service users” needs. However, health professionals have difficulty in coding health complaints, since they have not been specially trained to do so. This problem constitutes a flaw in the e-SUS/APS strategy, as its manuals do not provide details on the use of ICPC-2.

We conclude that (i) the greater number of user embracement records with reasons classified as “procedures”, and (ii) “General and unspecified” reasons, as per ICPC-2 (although this came in fifth place) ratify the need for improvements in the use of ICPC-2 in primary health care practice, in order to more adequately characterize the population's demand for services. In this sense, before the completion of this study, the municipal health department conducted a review of the user embracement protocol, including information related to the ICPC-2, and developed new continuing education activities with the professionals working in services providing user embracement, reiterating the need for the proper use of this classification. Understanding the reasons for user embracement, according to the profile of use, enables health services to better organize themselves to meet the needs of the population.
